# Protective effects of synbiotic soymilk fortified with whey protein concentrate and zinc sulfate against bile duct ligated-induced hepatic encephalopathy 

**Published:** 2020

**Authors:** Yahya Jalilpiran, Nader Tanideh, Samane Rahmdel, Negar Azarpira, Maral Mokhtari, Zohreh Mazloom

**Affiliations:** 1 *Department of Clinical Nutrition, School of Nutrition and Food Sciences, Shiraz University of Medical Sciences, Shiraz, Iran*; 2 *Students’ Scientific Research Center (SSRC), Tehran University of Medical Sciences (TUMS), Tehran, Iran*; 3 *Stem Cell and Transgenic Technology Research Center, Shiraz University of Medical Sciences, Shiraz, Iran*; 4 *School of Nutrition and Food Sciences, Shiraz University of Medical Sciences, Shiraz, Iran*; 5 *Transplant Research Center, Shiraz University of Medical Sciences, Shiraz, Iran*; 6 *Department of Pathology, School of Medicine, Shiraz University of Medical Sciences, Shiraz, Iran*

**Keywords:** Hepatic encephalopathy, Soymilk, Synbiotic, Whey protein, Zinc sulfate

## Abstract

**Aim:**

This study aimed to compare the effects of synbiotic soymilk fortified with whey protein concentrate and zinc sulfate with lactulose on bile duct ligated-induced HE.

**Background:**

Hepatic encephalopathy (HE) is seriously associated with neuromuscular and cognitive alterations.

**Methods:**

Eighty-two Sprague Dawley rats were randomly assigned into seven groups (sham, bile duct ligation (BDL), BDL + lactulose, BDL + soymilk (SM), BDL + Synbiotic soymilk (SSM), BDL + SSM + whey protein concentrate (WPC), BDL + SSM + WPC + ZnSO4). Different SM products, lactulose, and normal saline were administered via oral gavage (2 mL/rat/day). The serum and liver markers as well as liver histopathology were assessed after 28 days.

**Results:**

The SM products significantly reduced the serum alanine aminotransferase, albumin, and ammonia (*P* < 0.05). The levels of aspartate aminotransferase, endotoxin, and liver interleukin-6 improved significantly in all treatments, except for those receiving SM. SSM and SSM + WPC + ZnSO4 were the only effective products in reducing serum alkaline phosphatase (*P* < 0.05). Furthermore, the liver total antioxidant capacity was greater (*P*<0.05) in the SSM + WPC and SSM + WPC + ZnSO4 groups. The histopathological examinations confirmed the efficiency of all SM products in reducing liver fibrosis. Liver bile duct proliferation diminished only in the SSM + WPC and SSM + WPC+ ZnSO4 groups (P<0.05).

**Conclusion:**

This study showed the positive effects of different SM products, especially SSM + WPC and SSM + WPC + ZnSO4, on HE. Further studies are required to confirm our findings.

## Introduction

 In the last few years, there has been growing interest in the study of serious neuropsychiatric syndrome, known as hepatic encephalopathy (HE), which results from an acute/chronic liver disease and portosystemic shunting progression (1). The syndrome is characterized by a wide range of neuropsychiatric and motor disturbances, ranging from neuromuscular and cognitive alterations to coma and even death (2). Although early studies have regarded gut-derived neurotoxins (especially ammonia) as the main contributors to the HE pathogenesis, it has recently been demonstrated that multi-organ dysfunctions and many neurotransmitter systems, such as the monoaminergic, opioidergic, and gamma-aminobutyric acid (GABA)-ergic (3-5) mechanisms are also involved in HE. In other studies, the severity of HE has been shown to be correlated with the plasma levels of pro-inflammatory cytokines  (6, 7), which also highlights the critical role of the systemic inflammatory response in this disease. 

Lactulose and rifaximin are currently the main interventions in HE management (8, 9). Due to some gastrointestinal disturbances (nausea, abdominal cramps, and anorexia), lactulose is not well-tolerated by patients  (10). Rifaximin is also used to reduce the risk of overt hepatic encephalopathy (OHE) recurrence; however, it is not effective enough to alleviate all major symptoms of HE (11, 12). Alternative therapies, such as probiotics, have therefore attracted more attention over recent years (13). This interest has been motivated by the fact that HE is associated with gut dysbiosis and endotoxemia, which are in turn linked to systemic and cerebral inflammation (7, 14-16). Probiotics are thought to have several beneficial effects on HE, as their usage has proved to be correlated with a decline in oxidative stress, pro-inflammatory cytokines, and blood ammonia (17). 

Probiotic microorganisms are susceptible to degradation by gastric acid and their passage across the gastrointestinal tract; therefore, they need a carrier system (18). In this regard, soymilk seems to be a suitable choice (19, 20). The probiotic-enriched soymilk can be considered a synbiotic product because of its oligosaccharide content (21, 22). Further, plant proteins have proved to be well tolerated by HE patients compared to the animal origin proteins  (23, 24). In spite of such beneficial effects, consumption of soy products may have some limitations such as undesirable beany flavor and gastrointestinal discomforts, due to their non-digestible oligosaccharide constitutes      (25). 

Fermentation is a promising solution to diminish such limitations. It can also improve the safety and antioxidant activity of soy products (26-28). In addition, the bioavailability of soy isoflavones increases following fermentation (29). The other strategy to promote probiotic growth and stability during storage and support probiotic survival during passage through the digestive tract is to incorporate prebiotics, such as inulin, into probiotic foods (30-32). 

Both probiotics and nutrients, such as branched-chain amino acids (BCAAs), have been reported to be effective in HE treatment (33-36). Low levels of BCAAs or insufficient dietary protein intake have been proposed to worsen the complications of HE (37). Due to the high content of BCAAs in them, as well as their anabolic, antioxidant, and anti-inflammatory properties, whey proteins are considered as interesting candidates for HE management (38-41).

Zinc deficiency is another risk factor for HE as an inverse association has been reported between the serum levels of zinc and ammonia (42-44). Poor dietary zinc intake due to protein-restricted diet, increased urinary excretion, and disturbed zinc absorption by intestine are the most potent factors for zinc deficiency in HE (45). Both animal (46-48) and human studies (46, 47, 49) have also shown some beneficial effects for zinc supplementation in either hyper-amniotic or HE conditions.

Accordingly, the present study aimed to evaluate the effects of the following soymilk products on the blood and liver markers of rats with bile duct ligated (BDL)-induced HE: 1. soymilk (SM), 2. synbiotic soymilk (SSM), 3. SSM fortified with whey protein concentrate (WPC), and 4. SSM fortified with WPC and zinc sulfate (ZnSO4). 

## Methods

**Preparation and characterization of soymilk products**

***Preparation***

SM preparation was performed according to the method described previously (46, 47). In brief, soybeans (250 gr) were soaked in water overnight. After decanting the water, they were mixed with distilled water at a ratio of 1:3 (w/v) using a commercial kitchen blender unit equipped with an Eastman Tritan copolyester jug and stainless-steel blades (JTC Electronics Corp., OmniBlend I series, Model TM-767, Guangdong, China). Then, the mixture was filtered through double-layered cheesecloth to produce SM. The other products, including SSM, SSM fortified with WPC, and SSM fortified with WPC and ZnSO4, were prepared as follows.

Inulin (Merck, Darmstadt, Germany) was added to SM at a concentration of 2% (w/v). The mixture was pasteurized for 30 min at 85 °C and then divided into three equal portions. Two portions were supplemented with pasteurized WPC solution (60 °C for 30 min) to a final concentration of 7.5% (w/v). Three preparations were then inoculated with 0.01% (w/v) of commercial lyophilized *Lactobacillus* *acidophilus* La-5 (*L. acidophilus*; Chr. Hansen, Hørsholm, Denmark) and incubated at 37 °C until a pH of 4.70 was reached. Afterward, one of the WPC-containing fermented products was fortified with ZnSO4 at 800 mg/L. All products were cooled to 5 ᵒC and stored at 4 °C for up to 7 days.

***Physicochemical analysis***

The pH was determined at about 25 °C using a Metrohm Model 827 pH meter (Metrohm Ltd., Herisau, Switzerland). Total titratable acidity (TTA) was measured by titrating samples with 0.1 N NaOH using a phenolphthalein indicator. The total solids content was evaluated via air oven drying at 105±1 °C (Memmert, Schwabach, Germany). The percentage of soluble solids (°Brix) was obtained using a handheld refractometer model MT-098 (Three-In-One Enterprises Co., Taipei, Taiwan). The ash content was determined by dry-ashing in a muffle furnace regulated to 550 °C. The total fat was determined by Soxhlet extraction (50).

***Antioxidant capacity of products***

The Ferric Reducing Antioxidant Power (FRAP) assay was used to estimate the antioxidant capacity of products based on Benzie and Strain’s method (51). FRAP reagent was prepared by mixing 2.5 mL of 10 m*M* TPTZ (2,4,6-tripyridyl-s-triazine) solution in 40 m*M* hydrochloric acid, 2.5 mL of 20 m*M* ferric chloride solution, and 25 mL of 300 m*M* sodium acetate buffer (pH 3.6). The aqueous extract of the samples was obtained through 10-min centrifugation at 10000 ×g. Next, 1 mL aliquot of the diluted extract was mixed with 3 mL of FRAP reagent. After 5 min at 37 °C, the absorbance was measured against a reagent blank at 593 nm using a UV–visible spectrophotometer (Metrolab 330, Buenos Aires, Argentina). The standard curve was prepared using 0.26-8.8 mg/100 mL solutions of ascorbic acid. The results were expressed as mg ascorbic acid equivalent (AAE)/100 g.

***Microbiological analysis***

The microbiological quality of the samples was investigated on days 1 and 7. On each test day, the serial dilutions of each sample were prepared in a solution of 0.9% NaCl (w/v). *L. acidophilus* was counted on de Man, Rogosa, and Sharpe (MRS, Merck) agar incubated anaerobically at 37 °C for 72 hr. Yeast extract glucose chloramphenicol (YGC, Merck) agar was used to enumerate yeasts and molds. The total coliforms were counted using violet red bile lactose agar (VRBLA; Merck) at 35 °C for 24 hr (52). The samples were also examined for the presence of coagulase-positive staphylococci and *Escherichia coli* (*E. coli*) based on the procedure outlined by US FDA (53) and Iranian national standard-No 5234 (54), respectively.

**Animals and experimental design**

***Animals***

Eighty-two male Sprague-Dawley rats (220 ± 20 g weight) were purchased from the Centre for Comparative and Experimental Medicine, Shiraz University of Medical Sciences, Shiraz, Iran. They were adapted to the laboratory environment for one week prior to the experiment and received the chow diet and *ad libitum* drinking water during the study. The animals were kept at 22 ± 2 °C  , under a relative humidity of 50%, and a 12 hr light/dark cycle, in plastic cages on hardwood bedding. They received human care in compliance with the Guidelines for the Care and Use of laboratory animals approved by the ethics committee of Shiraz University of Medical Sciences, Shiraz, Iran. This study was approved by the ethics committee of Shiraz University of Medical Sciences, Shiraz, Iran (# 95-01-84-12821).

***HE Induction***

Following anesthetization by intramuscular injection of ketamine hydrochloride (100 mg/kgbw) and xylazine (5 mg/kgbw), a midline incision was made, and the common bile duct was identified, doubly ligated, and divided (55). In the sham-operated rats, the identified common bile duct was manipulated without any ligation. 

***Experimental setup***

One day after the operation, the rats were assigned into seven groups as follows: A) sham-operated rats (SH) (n = 10); B) BDL-rats (BDL) (n = 12); C) BDL-rats receiving lactulose (BDL + L) (n = 12); D) BDL-rats receiving SM (BDL + SM) (n = 12); E) BDL-rats receiving SSM (BDL + SSM) (n = 12); F) BDL-rats receiving SSM + WPC (BDL + SSM + WPC) (n = 12); and G) BDL- rats receiving SSM + WPC + ZnSO4 (BDL + SSM + WPC + ZnSO4) (n = 12). The soymilk products and L were administered to the rats by gavage technique at a level of 2 mL/rat/day. Groups A and B also received 2 mL/rat/day normal saline. Four weeks post-intervention, the rats were fasted for 12 hr and anesthetized with ketamine hydrochloride (100 mg/kg) plus xylazine (5 mg/kg). The blood (from cardiac puncture) and liver samples were collected for further analysis. The blood samples were centrifuged at 3000 ×g for 15 min for serum separation. The serum and liver samples were kept at −80 °C   until further analysis.

**Analysis of blood and liver parameters**

***Blood biochemistry markers***

The serum levels of alanine aminotransferase (ALT), aspartate aminotransferase (AST), alkaline phosphatase (ALP), albumin (ALB), and total bilirubin (TB) were measured using standard commercial kits (Pars Azmun Co. Tehran, Iran). Serum ammonia (SA) was determined using an ammonia assay kit (Ziest Chem Diagnostics, Tehran, Iran). All parameters were analyzed using an auto-analyzer (BT-1500; Biotecnica instruments, Rome, Italy). The endotoxin level was measured by the Enzyme Linked Immunosorbent Assay (ELISA) using a ZellBio kit (Germany).

***Hepatic total antioxidant capacity and IL6 levels***

The liver samples (500 mg) were homogenized in phosphate buffered saline (pH 7.0, containing 0.25 *M* sucrose); the supernatant was collected by centrifugation at 12000 ×g for 30 minutes. The samples were then analyzed for the total antioxidant capacity (TAC) and IL6 content by spectrophotometry (Pars Azmun Co, Tehran, Iran) and ELISA (Zellbio, Germany), respectively.

***Liver tissue histopathology***

To perform the histopathological examinations, the liver samples were fixed in buffered formalin solution (0.4% NaH_2_PO_4_, 0.64% Na_2_HPO_4_, and 10% formaldehyde in distilled water). The paraffin-embedded sections of the tissue (5 µm) were prepared and stained with hematoxylin and eosin (H&E) prior to light microscopic analysis. Masson’s trichrome staining was also used to identify liver fibrotic changes. The scores of the bile duct proliferation, inflammation, and liver fibrosis were evaluated according to Ara et al. (56).

**Statistical analysis**

The results were analyzed using IBM SPSS 22.0 statistical software (IBM Corp., Armonk, NY, USA). The results were shown as Mean ± SD and evaluated by the one-way analysis of variance (ANOVA) followed by the post hoc Duncan’s multiple range tests. The Mann-Whitney and paired Wilcoxon tests were used to compare the probiotic count of different soymilk products. *P* values lower than 0.05 were considered statistically significant. 

**Table 1:** Physicochemical parameters of soymilk products

Soymilk products	pH	TTA (% lactic acid)	Solids content (%)	SSC (°Brix)	Ash (%)	Fat (%)	TAC (mg AAEs/100 g (
SM	6.50 ± 0.14	0.16 ± 0.02	6.25 ± 0.35	4.00 ± 0.00	1.56 ± 0.02	1.27 ± 0.05	16.14 0.23
SSM	4.73 ± 0.01	0.39 ± 0.05	7.00 ± 0.70	3.00 ± 0.00	1.97 ± 0.10	1.16 ± 0.02	18.16 0.23
SSM + WPC	4.70 ± 0.07	1.10 ± 0.02	13.00 ± 0.70	8.75 ± 0.35	2.07 ± 0.24	1.34 ± 0.05	34.14 0.22
SSM + WPC + Znso_4_	4.74 ± 0.05	1.41 ± 0.07	12.50 ± 0.00	8.50 ± 0.00	2 .22 ± 0.74	1.30 ± 0.04	34.73 0.25

**Table 2:** Probiotic count of different soymilk products

Soymilk products	*L. acidophilus* count (log_10_ cfu mL^-1^)	
	Day 1	Day 7
SM	----	----
SSM	8.67 ± 0.26^Aa^	8.06 ± 0.65^Ba^
SSM + WPC	9.32 ± 0.35^b^	9.32 ± 0.24^b^
SSM + WPC + Zn${\mathrm{so}}_{4}$	9.40 ± 0.40^b^	9.34 ± 0.26^b^

Values are given as mean ± standard deviation of three replicates; Means in the same row with different capital letters indicate signiﬁcant differences (*P*< 0.05) between storage days for each treatment; Means in the same column with different lowercase letters indicate signiﬁcant differences (*P*< 0.05) between treatments at the same time; SM, soymilk; SSM, synbiotic soymilk; WPC, whey protein concentrate.

**Table 3:** Liver function tests and serum ammonia levels after four weeks of treatment

Groups	N	ALT ( U/L )	AST (U/L)	ALP (U/L)	TB (mg/dl)	DB (mg/dl)	Alb (mg/dl)	SA (µg/dl)
Sham	10	30 ± 6 ^a^	79 ± 8^ a^	535 ± 167 ^a^	0.40 ± 0.00 ^a^	0.09 ± 0.00 ^a^	3.38 ± 0.23 ^a^	210 ± 25 ^a^
BDL	9	424 ± 88 ^b^	868 ± 187 ^b^	1844 ± 376 ^b^	5.94 ± 0.91 ^b^	4.89 ± 0.61 ^b^	2.81 ± 0.46 ^b^	646 ± 95 ^b^
BDL + L	9	419 ± 105 ^b^	658 ± 285 ^c, d^	1618 ± 396 ^b, c, d^	5.68 ± 0.31 ^b^	4.66 ± 0.40 ^b^	2.98 ± 0.55 ^b, c^	374 ± 93 ^c, d^
BDL + SM	9	244 ± 79 ^c^	774 ± 94 ^b, c^	1694 ± 285 ^b, c^	5.83 ± 0.22 ^b^	4.90 ± 0.41 ^b^	3.24 ± 0.36 ^c, d^	440 ± 132 ^c^
BDL + SSM	10	211 ± 101 ^c^	662 ± 125 ^c, d^	1503 ± 183 ^c, d^	5.87 ± 0.61 ^b^	4.82 ± 0.43 ^b^	3.19 ± 0.38 ^c, d^	403 ± 127 ^c, d^
BDL + SSM + WPC	10	218 ± 61 ^c^	600 ± 173 ^d^	1605 ± 170 ^b c, d^	5.50 ± 0.56 ^b^	4.48 ± 0.72 ^b^	3.24 ± 0.23 ^c, d^	328 ± 76 ^d^
BDL + SSM + WPC+ Zn	10	186 ± 53 ^c^	676 ± 129 ^c, d^	1386 ± 112 ^d^	5.57 ± 1.11^ b^	4.66 ± 1.00 ^b^	3.27 ± 0.24 ^c, d^	379 ± 110 ^c, d^

BDL, bile duct ligation; L, lactulose; SM, soymilk; SSM, synbiotic soymilk; WPC, whey protein concentrate; ALT, alanine aminotransferase; AST, aspartate aminotransferase; ALP, alkaline phosphatase; TB, total bilirubin; Alb, albumin; SA, serum ammonia. Data are presented as mean ± SD. Means in the same column with different lowercase letters represent signiﬁcant differences (*P*< 0.05) between treatments.

## Results

**Characterization of soymilk products**

The mean values of the physicochemical properties of SM products are shown in Table 1. All the samples were negative for yeasts and molds, coliforms, coagulase-positive staphylococci, and *E. coli*. The *L. acidophilus* concentration of WPC-fortified products was significantly higher than that of the SSM (Table 2). In all synbiotic samples, however, the probiotic count remained above 10^8^ cfu mL^-1^ over the seven days of storage at 4 °C.

**Blood biochemistry markers**

**Figure 1:**
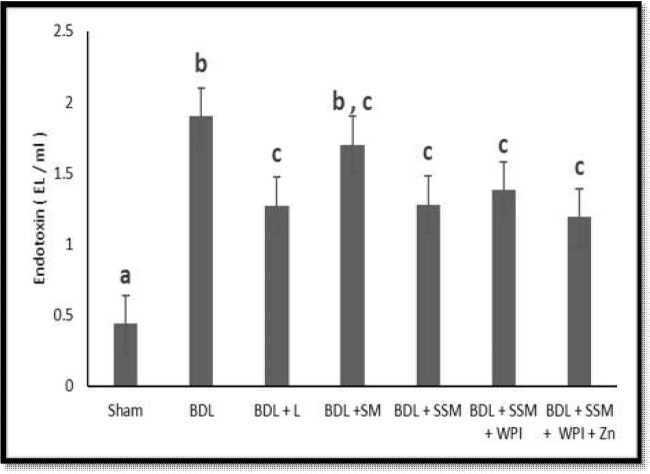
Effect of different treatments on serum endotoxin levels; BDL, bile duct ligation; L, lactulose; SM, soymilk; SSM, synbiotic soymilk; WPC, whey protein concentrate. Data are presented as mean ± SD. Different lowercase letters indicate signiﬁcant differences (*P*< 0.05) between treatments

During the study, 15 rats from the experimental groups were expired and excluded from the analysis. The effects of L and different SM products on liver function tests (ALT, AST, ALP, TB, and ALB), serum ammonia, and endotoxin are summarized in Table 3. The sham-operated and BDL groups exhibited significant differences among their respective serum levels of ALT, AST, ALP, TB, and ALB. Compared to the BDL group, the‏ SM-product treated groups revealed significantly lower levels of ALT while L did not show any significant effect. All treatments, with the exception of SM, significantly reduced the serum AST concentration compared to the BDL group. The SSM and SSM + WPC + ZnSO4 products were the only treatments that significantly reduced the ALP levels. Except in the L group, all the SM treatments led to a significant rise in the serum concentration of ALB. No significant differences were found between the TB levels of different treatments. In the BDL group, the serum ammonia concentration significantly increased compared to that of the sham-operated group. All the treatments significantly lowered the serum ammonia levels (with the greatest and lowest reductions observed in the BDL + SSM + WPC and BDL + SM groups, respectively). Furthermore, the serum endotoxin levels significantly increased in the BDL group compared to those of the rats in the sham-operated group. In contrast with the BDL group, all the treatments, except for the SM, had significantly lower levels of serum endotoxin (Figure 1). 

**Hepatic total antioxidant capacity and IL6 levels**

**Figure 2:**
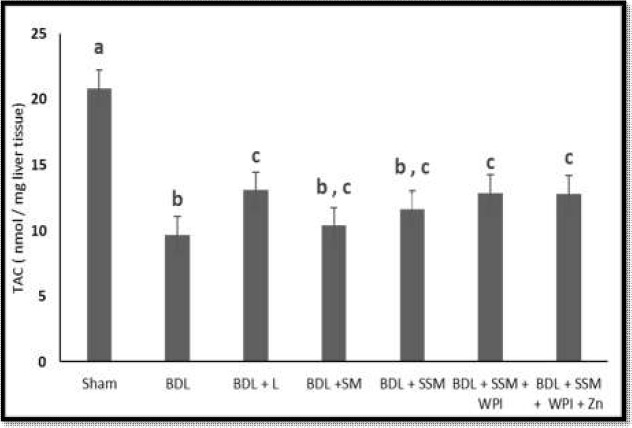
Effect of different treatments on liver total antioxidant capacity levels; BDL, bile duct ligation; L, lactulose; SM, soymilk; SSM, synbiotic soymilk; WPC, whey protein concentrate. Data are presented as mean ± SD. Different lowercase letters reveal signiﬁcant differences (P< 0.05) between treatments

**Figure 3:**
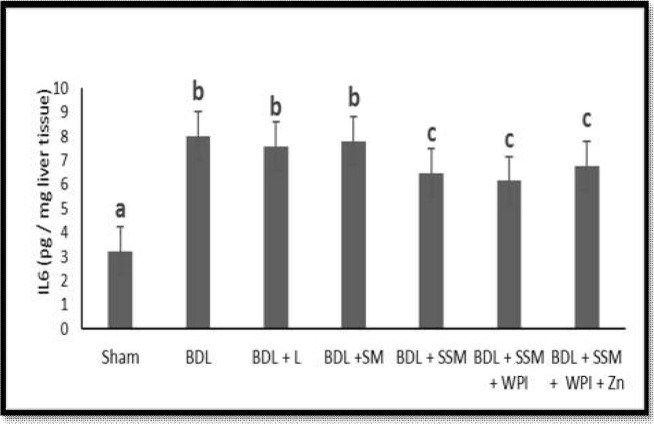
Effect of different treatments on Liver IL6 levels; BDL, bile duct ligation; L, lactulose; SM, soymilk; SSM, synbiotic soymilk; WPC, whey protein concentrate. Data are presented as mean ± SD. Different lowercase letters represent signiﬁcant differences (P< 0.05) between treatments

The effects of different treatments on liver TAC and IL6 levels are shown in Figures 2 and 3**,** respectively. In the BDL group, the TAC concentrations significantly decreased while the IL6 increased compared to that of the sham-operated group. Treatment with L, SSM + WPC, and SSM + WPC + ZnSO_4_ significantly elevated the TAC levels compared to those of the BDL group. The liver levels of IL6 dropped in the BDL + SSM, BDL + SSM + WPC, and BDL + SSM + WPC + ZnSO_4_ groups in comparison with those of the BDL group (𝑃 < 0.05).

**Liver tissue histopathology**

**Figure 4:**
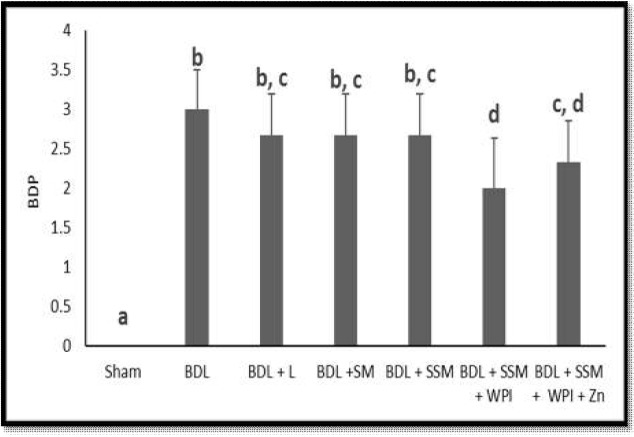
Effect of different treatments on Bile duct proliferation (BDP) (n = 6); BDL, bile duct ligation; L, lactulose; SM, soymilk; SSM, synbiotic soymilk; WPC, whey protein concentrate. Data are presented as mean ± SD. Different lowercase letters indicate signiﬁcant differences (P< 0.05) between treatments

**Figure 5:**
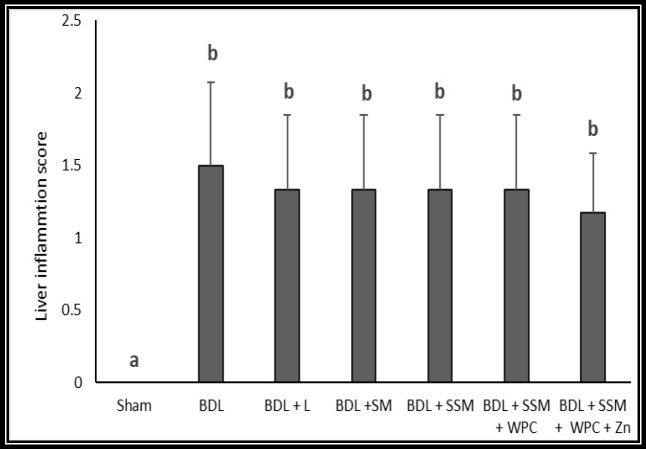
Effect of different treatments on Liver inflammation score (n = 6); BDL, bile duct ligation; L, lactulose; SM, soymilk; SSM, synbiotic soymilk; WPC, whey protein concentrate. Data are presented as mean ± SD. Different lowercase letters reveal signiﬁcant differences (P< 0.05) between treatments

**Figure 6:**
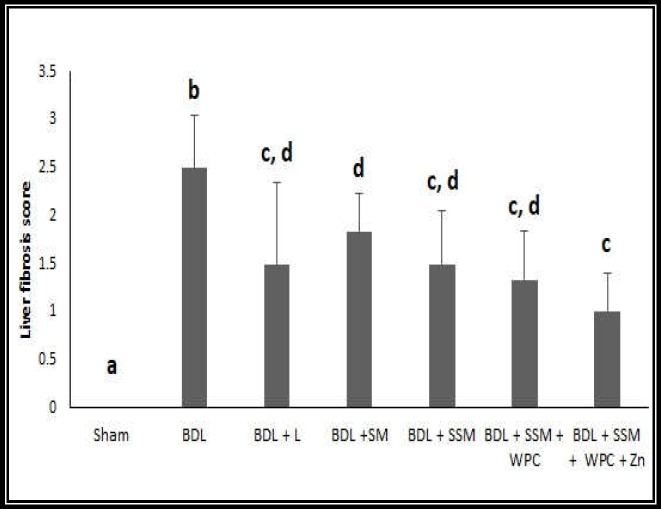
Effect of different treatments on Liver fibrosis score (n = 6); BDL, bile duct ligation; L, lactulose; SM, soymilk; SSM, synbiotic soymilk; WPC, whey protein concentrate. Data are presented as mean ± SD. Different lowercase letters show signiﬁcant differences (*P*< 0.05) between treatments

Liver histopathological changes in the BDL rats revealed an extensive bile duct proliferation (BDP), inflammation, and fibrotic changes of tissue (Figures. 4, 5, and 6). BDP was significantly inhibited in the BDL rats following treatment with SSM + WPC and SSM + WPC + ZnSO4. Liver fibrosis also diminished in all treatment groups; the greatest reduction was found in the SSM + WPC + ZnSO4 group. There was no significant difference between all treatment groups and the BDL group regarding changes in liver inflammation. The legend figures. 1 and 2 indicate the histopathological photomicrographs of the liver in bile duct ligated rats treated with lactulose and different soymilk products obtained from hematoxylin and eosin and Masson’s trichrome staining, respectively. Based on the hematoxylin and eosin staining, the septal ductular proliferation grade (2) was noticed in the BDL+ L, BDL + SM, and BDL + SSM groups. It also showed that the ductular proliferation was limited to the portal area in group E. Furthermore, Masson’s trichrome staining revealed that liver fibrosis was limited to the portal area in group F. In other groups, the fibrosis score also declined compared to that of the BDL group.

## Discussion

**Legend Figure 1:**
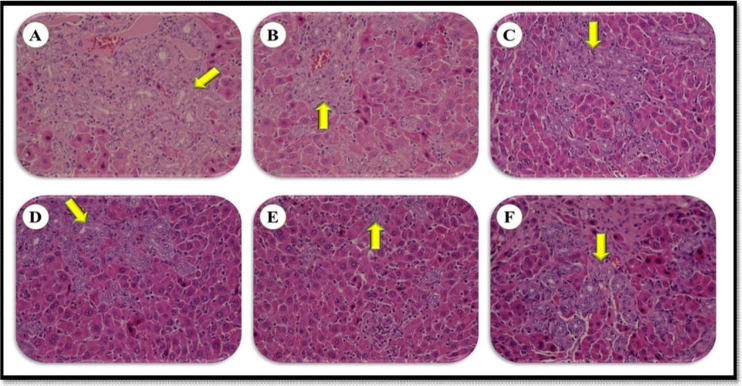
The histopathological photomicrographs of the liver in bile duct ligated rats treated with lactulose and different soymilk products; BDL (A), BDL + L (B), BDL + SM (C), BDL + SSM (D), BDL + SSM + WPC (E), BDL + SSM + WPC + ZnSO4 (F). Septal ductular proliferation grade (2) was noticed in groups B, C, D. In group E, ductular proliferation was limited to the portal area (refer to Fig. 4) (H & E × 400). BDL, bile duct ligation; L, lactulose; SM, soymilk; SSM, synbiotic soymilk; WPI, whey protein concentrate

**Legend Figure 2:**
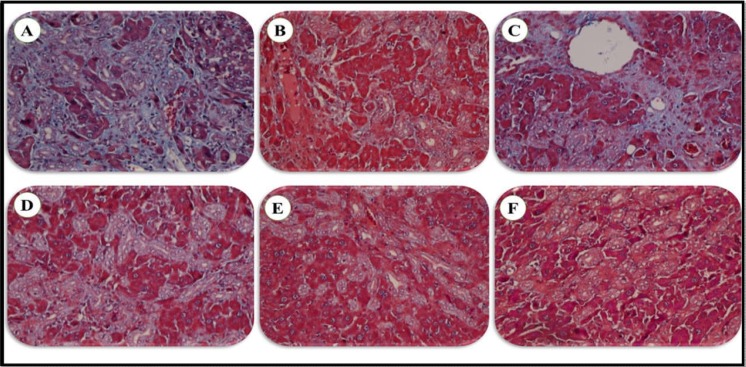
The histopathological photomicrographs of the liver in bile duct ligated rats treated with lactulose and different soymilk products; BDL (A), BDL + L (B), BDL + SM (C), BDL + SSM (D), BDL + SSM + WPI (E), BDL + SSM + WPI + ZnSO4 (F). In group F, liver fibrosis was limited to the portal area. In other groups the fibrosis score also decreased compared to the BDL group (A) (refer to Fig. 6) (Masson’s trichrome × 400). BDL, bile duct ligation; L, lactulose; SM, soymilk; SSM, synbiotic soymilk; WPC, whey protein concentrate

The BDL is a desirable model in studying liver cirrhosis as its histological and biochemical changes resemble those of the secondary biliary cirrhosis in humans (57). In the present study, the hepato-protective effect of different SM products on the bile duct ligated-induced HE was compared to that of the lactulose in the rats.

Compared to the lactulose, the SM products were more effective in improving the serum ALB and ALT. However, they did not have any priority over each other. The main contributing factor, thus, seems to be soymilk. The efficiency of soy and its products in reducing ALT has been previously reported (58, 59); this effect can be mainly attributed to soy bioactive compounds, especially isoflavones (60, 61). Limited data, however, are available on the potential role of soy and its products in improving the serum Alb level (60, 62). As described by other researchers (59), fermentation cannot improve the efficiency of soy in reducing ALT. Further, there are several human and animal studies suggesting the inefficiency of zinc (63) and whey proteins (64) in reducing ALT. 

 There are some data available in the literature regarding the beneficial effect of fermented soy products on AST reduction (59). In the current study, the SSM products were as effective as lactulose in reducing serum AST and endotoxin levels. However, the SSM products were the only effective treatments in reducing IL6. Given the inefficiency of soymilk in improving these markers, the synbiotic formulation of probiotic and inulin seems to be the main contributor. The protective effects of a combination of *L. acidophilus* and inulin against Salmonella-induced liver damage through the reduction of serum ALT and AST levels have been reported by Rishi et al. (65). Such influence of probiotics and synbiotics has well been documented in a meta-analysis on 17 human trials (66). The lowering effects of probiotic and synbiotic soymilk products on inflammatory markers, including serum lipopolysaccharides (LPS), serum, and liver tumor necrosis factor-α (TNF- α), have been suggested by several researchers (67, 68). These products have proved to modulate the GI microbiota and, thus, decrease endotoxemia (21, 65, 69). In addition, probiotic and synbiotic formulations have the potential to reduce the bacterial translocation and its related marker(s) in HE (IL6) (70-72).

The serum levels of ALP significantly decreased following treatment with SSM and SSM + WPC + ZnSO4. In the study conducted by Lin et al. (73), an eight-week administration of fermented soymilk powder to rats led to a significant reduction in the serum ALP. Further, the impact of zinc supplementation on the reduction of this factor in a rat model of liver fibrosis has been previously reported (63). In a study by Jobara et al., it was revealed that the administration of a whey-hydrolyzed peptide-enriched immunomodulating diet to cirrhotic rats could improve the serum levels of the liver enzymes in those rats (74).

Although the SM products were not statistically more effective than lactulose in reducing serum ammonia in the present study, the minimum level was observed in the group treated with SSM + WPC. Soymilk and WPC are both rich in branched-chain amino acids (75, 76), which have been reported to be effective in HE treatment (33, 34, 36). These amino acids are able to stimulate hepatic protein synthesis through stimulating hepatocyte growth factor production by stellate cells (77). A recent study has shown that milk-derived alpha-lactalbumin, as the main component of whey protein, could effectively lower the serum ammonia level in a rat model of thioacetamide-induced liver and brain damage (78). Further, the fiber content of soybeans can increase the GI movements which will, in turn, reduce nitrogen absorption, increase nitrogen excretion, and change colonic microbiota (79, 80). The efficiency of zinc-containing products in reducing ammonia might be due to the role of zinc in nitrogen metabolism which functions based on the direct action of zinc on the enzymes, including urea cycle enzymes in the liver and glutamine synthetase in the muscle tissue, contributing to the regulation of blood ammonia levels (42, 63). The oral zinc supplementation has proved to be effective in reducing blood ammonia and increasing liver ornithine transcarbamylase activity in carbon tetrachloride-induced cirrhosis (63). 

Higher levels of liver TAC and lower BDP scores were found in rats treated with SSM + WPC and SSM + WPC + ZnSO4 products. These products had the highest antioxidant power. The antioxidant potential of ZnSO4 supplementation was observed through the improvement of antioxidant enzymes activity, such as catalase and malondialdehyde levels, as already reported in the rat model of liver cirrhosis (81). The latter treatment was not, however, more effective than the former, indicating the crucial role of whey proteins. There is growing evidence on the anabolic, antioxidant, and anti-inflammatory properties of whey proteins (82-84). More specifically, the effects of whey proteins and their derivatives on the liver TAC levels have been documented in several animal studies (85, 86). A possible explanation for such observations is the contribution of cysteine to glutathione synthesis. Glutathione is an antioxidant and anti-carcinogenic tri-peptide which provides protection against oxidant-induced cell damage (87, 88). 

There is a growing interest in the health benefits of soy isoflavones, such as genistein, genistein, daidzin, and daidzein, whose role in the prevention of liver fibrosis and injury has been suggested previously (89, 90). The results of this study indicated the anti-fibrotic effects of soymilk products. However, the most effective soymilk product was SSM + WPC + ZnSO_4_. Several studies have shown the protective effects of zinc supplementation against ischemia-reperfusion-induced liver injury (91). In another study, the intra-gastric administration of ZnSO_4 _prevented the progression of liver fibrosis in the mice model of BDL through inhibiting collagen production and/or deposition, and the increase in collagen degradation (92). A 3-week treatment with whey-hydrolyzed peptide-enriched immunomodulating diet was reported to be effective in preventing liver fibrosis (74). There is also some evidence on the hepato-protective effects of WPC supplementation against tetrachloride-induced liver fibrosis in rats (93). Nevertheless, the ameliorating effects of soymilk products found in the current study could be at least partially attributed to the synergistic relationship between WPC and ZnSO4. In a study conducted by Hayashi et al.  (94), it was found that a combination of BCAAs and zinc was more effective than the single compounds in managing liver cirrhosis. 

## Conclusion

The results of this study revealed that synbiotic soymilk fortified with whey protein concentrate could improve serum and liver markers and reduce the development of HE. The underlying molecular mechanism may cause some alterations in the levels of serum LPS, liver IL6, and liver TAC. It, therefore, can be suggested that soymilk has the potential to be used as an alternative therapy for HE management. Although the greatest beneficial effects were recorded following treatment with SSM + WPC + ZnSO4, the results were not statistically significant compared to the SSM + WPC group. Further studies with a longer duration of treatment are required to confirm the findings of this study.
